# Mental health impact of the Russian-Ukraine war on Canadian residents with or without Ukrainian descent

**DOI:** 10.1192/j.eurpsy.2024.360

**Published:** 2024-08-27

**Authors:** A. Belinda, R. Shalaby, Y. Wei, V. I. O. Agyapong

**Affiliations:** ^1^Department of psychiatry, University of Alberta, Edmonton; ^2^Department of psychiatry, Dalhousie University, Halifax, Canada

## Abstract

**Introduction:**

War tends to produce fear. The devastating and traumatic occurrences of war can have both short- and long-term effects on the mental well-being of populations. Russia’s invasion of Ukraine indirectly affects all populations, especially individuals of Ukrainian descent.

**Objectives:**

To assess the mental health impact of the Russian invasion of Ukraine on Canadian residents who subscribed to ‘Text4Hope Ukraine’ program and to ascertain if there are differences in mental health impacts between those with and without Ukrainian descent.

**Methods:**

Canadians were invited to self-subscribe to the text messaging program. An online survey was used to collect sociodemographic, war-related, and clinical information; stress, resilience, likely anxiety disorder and likely depressive disorder from subscribers. Outcome measures included baseline scores using validated scales. Data were analyzed using SPSS Version 25. To examine the association of psychological problems with the sociodemographic and war-related factors, univariate analysis using the Chi-square/Fishers Exact test was performed with two-tailed significance (p ≤ .05). An independent sample t-test with two-tailed significance (p-value ≤ 0.05) was employed to assess the differences in the respective mean scores of the psychological problems across the two groups. The first group represents the participants who did not have citizenship or ancestors from Ukraine (NUk), while the second group represents the respondents are Ukrainian who either have previously held citizenship or have ancestors/family from Ukraine (Ukr). No imputation of missing data and reported data represents the complete responses

**Results:**

Study findings reflected prevalence of low resilience (59.7%), moderate to high stress (87.5%), likely Generalized Anxiety Disorder (45.8%) and likely Major Depressive Disorder (38.9%). Respondents who identified as female had a higher likelihood of presenting with low resilience (χ2(1) = 5.68, p = .02) and likely Generalized Anxiety Disorder (χ2 (1) = 4.85, p = .03) compared to male respondents. There was no statistically significant difference in the mean scores of the four psychological problems based on any of the variables that suggest Ukrainian descent or not (p>.05).

**Conclusions:**

War can have negative impacts on all populations irrespective of their location, or association of individuals with the impacted country. This study provides valuable insights into the mental health impact of the Russian invasion of Ukraine on a specific sample of Canadian residents who subscribed to the ‘Text4Hope Ukraine’ text messaging program. This information is relevant when planning mental health intervention for this population. Governments should target and provide adequate mental health and psychosocial support or interventions for global populations at risk during war.

**Disclosure of Interest:**

None Declared

